# Overexpression of *Arabidopsis NLP7* improves plant growth under both nitrogen-limiting and -sufficient conditions by enhancing nitrogen and carbon assimilation

**DOI:** 10.1038/srep27795

**Published:** 2016-06-13

**Authors:** Lin-Hui Yu, Jie Wu, Hui Tang, Yang Yuan, Shi-Mei Wang, Yu-Ping Wang, Qi-Sheng Zhu, Shi-Gui Li, Cheng-Bin Xiang

**Affiliations:** 1 School of Life Sciences, University of Science and Technology of China, Hefei, Anhui Province 230027, China; 2 Rice Research Institute, Anhui Academy of Agricultural Sciences, Hefei 230031, China; 3 Rice Research Institute, Sichuan Agricultural University, Chengdu 611130, China

## Abstract

Nitrogen is essential for plant survival and growth. Excessive application of nitrogenous fertilizer has generated serious environment pollution and increased production cost in agriculture. To deal with this problem, tremendous efforts have been invested worldwide to increase the nitrogen use ability of crops. However, only limited success has been achieved to date. Here we report that NLP7 (NIN-LIKE PROTEIN 7) is a potential candidate to improve plant nitrogen use ability. When overexpressed in *Arabidopsis*, *NLP7* increases plant biomass under both nitrogen-poor and -rich conditions with better-developed root system and reduced shoot/root ratio. *NLP7*–overexpressing plants show a significant increase in key nitrogen metabolites, nitrogen uptake, total nitrogen content, and expression levels of genes involved in nitrogen assimilation and signalling. More importantly, overexpression of *NLP7* also enhances photosynthesis rate and carbon assimilation, whereas knockout of *NLP7* impaired both nitrogen and carbon assimilation. In addition, NLP7 improves plant growth and nitrogen use in transgenic tobacco (*Nicotiana tabacum*). Our results demonstrate that NLP7 significantly improves plant growth under both nitrogen-poor and -rich conditions by coordinately enhancing nitrogen and carbon assimilation and sheds light on crop improvement.

Nitrogen (N) is one of the essential macronutrients for plant growth and crop productivity. To meet the increased demands for agricultural production, a vast amount of nitrogenous fertilizers were applied to soil worldwide to maximize crop yields^1^. Global use of N fertilizer amounted to 110 million metric tons in 2007. By the year 2050, it is projected to increase to between 125 and 236 million metric tons^2^. However, only an average of 30–50% of the applied N is taken up by the plant depending on the species and cultivar, with the remainder being lost through various pathways, leading to significant environmental pollution and ecological imbalance^3^. Therefore, it is a great challenge to accommodate the demands of the expanding world population by developing a highly productive agriculture, whilst at the same time preserving the quality of the environment. One of the possible solutions to this dilemma is to improve the N use efficiency (NUE) of crops.

The definition of NUE has been defined in various ways, but the simplest is the amount of plant yield in terms of either total biomass or grain yield per unit of applied fertilizer N^4^,^5^. Generally, NUE is an integration of N uptake efficiency (NUpE) and N utilization (assimilation) efficiency (NUtE)^4^. Therefore, understanding the mechanisms regulating these processes is important for crop N use improvement. Many genes have been studied for increasing the N assimilation in various plants during the past decades. Most of these genes were related to N uptake and primary assimilation, including nitrate and ammonia transporters (NRTs and AMTs), nitrate and nitrite reductase (NR and NiR), aminotransferases and dehydrogenases, glutamine synthetase (GS), and glutamate synthase (GOGAT). However, the ectopic expression of these genes have been shown to affect N uptake or individual enzyme activity involved in N metabolism, but few have showed phenotypic effect on NUE or other growth parameters to date^4^,^5^,^6^. For example, overexpression of the high affinity ammonium transporter gene *OsAMT1;1* in rice enhanced N uptake, but was also associated with a decrease in biomass^7^,^8^. Overexpression of *GS* increased GS activity in some instances, but the effects on N assimilation and plant biomass were inconsistent^5^. Constitutive expression of the tobacco NR-encoding genes *Nia2* and *Nia* in different plants showed no NUE phenotype under N-limiting conditions^9^,^10^. Overexpression of *OsPTR6*, a PTR/NRT1 (PEPTIDE TRANSPORTER/NITRATE TRANSPORTER 1) family gene, in rice increased plant growth at different N conditions but decreased NUE at high ammonium supply^11^. Although no successful commercial transgenic plants expressing these genes have been reported to date, recent research progresses in rice shed light on improving NUE of rice. Elevated expression of *OsPTR9* in rice plants enhanced ammonium uptake, promoted lateral root formation and increased grain yield^12^. Sun *et al*.^13^ found rice DEP1 (DENSE AND ERECT PANICLES 1) modulated N responses by interacting with the heterotrimeric G proteins. Rice plants carrying the dominant *dep1-1* allele showed N insensitive vegetative growth coupled with increased N uptake and assimilation, leading to improved grain yield and harvest index at moderate levels of N fertilization. In another study, Hu *et al*.^14^ found a single SNP (single-nucleotide polymorphism) in rice *NRT1.1B* contributed to nitrate-use divergence between rice subspecies, and *NRT1.1B*-*indica* could potentially increase the NUE of *japonica*.

A critical coordination exists between C and N metabolisms. Improving the plant NUE can be compromised unless there is sufficient C available^15^. On the one hand, N deficiency has great impacts on chloroplasts development, content of chlorophyll and amino acids as well as proteins crucial for C assimilation, such as Rubisco (ribulose-1,5-bisphosphate carboxylase/oxygenase) and PEPC (phosphoenolpyruvate carboxylase)^16^,^17^. On the other hand, N assimilation requires energy, reducing power and carbon skeleton produced by C assimilation. Increasing C supply promotes N uptake and assimilation^18^. NUE is improved in elevated CO_2_ environment^19^. Practically speaking, genetic manipulation of the crosstalk points between N and C assimilation appears to be one of the most efficient attempts at improving NUE^5^.

Considering the complex regulation of N and C assimilation, transcription factors (TFs) might be a more efficient approach for improving N use ability, because they have the capacity to modulate expression of a set of genes coordinately. Many TFs regulated by nitrate have been identified^5^,^20^. Ectopic expression of maize *Dof1* (*ZmDof1*) TF in *Arabidopsis* and rice leads to the up-regulation of multiple genes involved in C-skeleton production and increased N assimilation and plant growth under low-N conditions^21^,^22^. The paralogous GATA TFs GNC (GATA, nitrate-inducible, carbon-metabolism-involved) and CGA1/GNL (cytokinin-responsive GATA1/GNC-like) modulate N assimilation, chloroplast development and starch production^23^. In *Lotus japonicas*, a nodule inception (NIN) TF was found necessary for symbiotic N fixation^24^. *NIT2*, a homologue of the *Arabidopsis* NIN-like protein (NLP) genes in *Chlamydomonas*, was reported as a central regulator required for nitrate signaling and assimilation. Mutants of this gene in *Chlamydomonas* are not able to activate the expression of the genes required for nitrate assimilation, and they are unable to grow on nitrate as the sole N source^25^,^26^. Recently, NLP TFs in *Arabidopsis* were reported to play a central role in nitrate signalling^27^. One of the NLP TFs, NLP7, modulates nitrate sensing and metabolism and plays as an orchestrator of nitrate responses^28^,^29^. NRG2 (NITRATE REGULATORY GENE 2) was reported to mediate nitrate signaling and interact with NLP7 in *Arabidopsis*^30^. In our present work, we explored the function of NLP7 in N and C assimilation in both *Arabidopsis* and tobacco. We investigated the plant growth and N use under different nitrate conditions and analyzed the possible mechanisms of NLP7 in N and C assimilation. Our data imply that NLP7 is a potential candidate for improving the N fertilizer use ability of crops.

## Results

### NLP7 increases plant biomass under both N-limiting and -sufficient conditions

**Figure 1:**
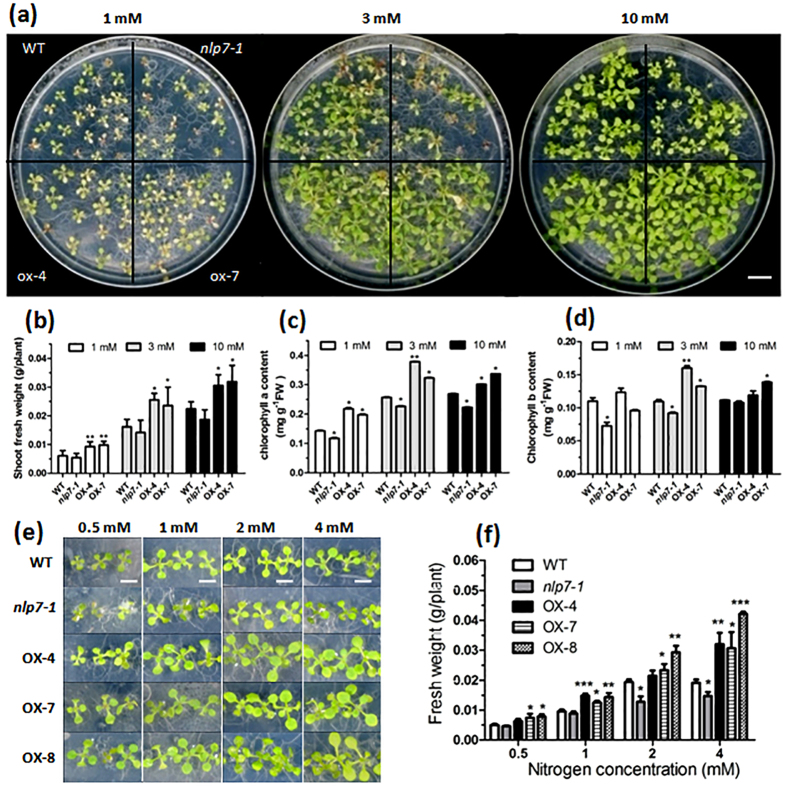
NLP7 improves plant growth under both nitrate-rich and -poor conditions. (**a**) The phenotypes of the 16-day-old WT, *nlp7-1* and *NLP7*-overexpressing plants grown on medium plates containing different concentrations of nitrate with a density of 80 plants per plate. Diameter of the plate is 14.5 cm. Bar = 1.7 cm. (**b**) Shoot fresh weight of the 16-day-old plants grown under different nitrate conditions. Values are the mean ± standard deviation (SD) of three independent replications each containing 20 plants per genotype (*P < 0.05, **P < 0.01). (**c–d**) The chlorophyll a (**c**) and b (**d**) contents of the 16-day-old plants. Values are the mean ± SD of three independent replications (*P < 0.05, **P < 0.01). (**e**) The phenotypes of the 10-day-old plants grown with a density of 28 plants per plate under different nitrate conditions. Bar = 0.5 cm. (**f**) Fresh weight of the 10-day-old plants under different nitrate conditions. Values are the mean ± SD of three independent replications each containing 20 plants per genotype (*P < 0.05, **P < 0.01, ***P < 0.001).

To investigate effects of *NLP7* expression level on plant growth, we generated 35S:NLP7 cDNA transgenic *Arabidopsis* plants and obtained a T-DNA insertion mutant *nlp7-1* (SALK_26134C) (Supplementary Fig. S1). Quantitative real-time PCR (qRT-PCR) analysis showed that the transcript levels of other *NLPs* had no significant difference in the *NLP7*-overexpressing and WT plants (Supplementary Fig. S2). Plants were grown on the modified MS (Murashige and Skoog) medium in which nitrate was used as sole inorganic N source. When grown on the medium containing different concentrations of nitrate, the *NLP7*-overexpressing plants showed obvious growth advantages compared with wild type (WT) and *nlp7-1* plants. The *nlp7-1* plants showed constitutive N-deficient phenotypes on both nitrate-rich and -poor media (Fig. 1a), which agreed with the previous study^29^. Statistical analyses indicated that overexpression of *NLP7* could increase biomass of the shoot under both nitrate-rich and -deficient conditions, and *NLP7* knockout impaired plant growth even under nitrate-rich conditions (Fig. 1b). We also found that the *NLP7*-overexpressing plants had higher chlorophyll contents than the mutant and WT plants under different nitrate conditions (Fig. 1c,d). These phenotypes were further proved by vertical growth assay as showed in Supplementary Fig. S3. Considering the influences of plant density on nitrate concentration, we grew 28 plants (7 plants in a row) per plate for further analysis. Results showed that the *NLP7*-overexpressing plants grew much better than the WT and *nlp7-1* plants on medium containing 0.5–4 mM nitrate, especially under the higher nitrate conditions (Fig. 1e). Compared with WT and *nlp7-1* plants, the *NLP7*-overexpressing plants had significantly increased fresh weight (FW) with the raising nitrate concentrations of the medium (Fig. 1f). Moreover, we conducted functional complementation analysis by expressing a functional pNLP7:NLP7-GFP fusion construct in the *nlp7-1* plants. The results showed that *nlp7-1* plants showed N-deficient phenotypes with lower FW and longer primary roots (PRs) on N-rich medium. However, these phenotypes could be restored by expressing pNLP7:NLP7-GFP construct (Supplementary Fig. S4). Taken together, these results demonstrate that the growth of *nlp7-1* is relatively insensitive to N level while the *NLP7*-overxpresssing plants improved growth and response to N availability.

Additionally, we also found that the *nlp7-1* plants displayed severe N-starved phenotypes with yellow leaves, while the *NLP7*-overexpressing plants still remained green after 3 days N starvation in liquid culture (Supplementary Fig. S5). Moreover, *NLP7* transgenic plants grew much bigger in N-limiting soil with significantly higher rosette surface area and rosette biomass than the WT and *nlp7-1* plants under short-day conditions. The mutant and WT plants showed much more severe N-deficient phenotypes with discolored rosette leaves compared with the *NLP7*-overexpressing plants (Supplementary Fig. S5). Overexpression of *NLP7* not only increased the shoot biomass, but also enhanced root growth with higher root biomass and longer roots, while *nlp7-1* mutant had much lower shoot and root biomass and delayed flowering compared with the WT (Supplementary Fig. S5). These results demonstrate that overexpression of *NLP7* in *Arabidopsis* enhanced the plant growth and N use as well as tolerance to N-deficiency.

### NLP7 alters root architecture and shoot/root ratio

**Figure 2:**
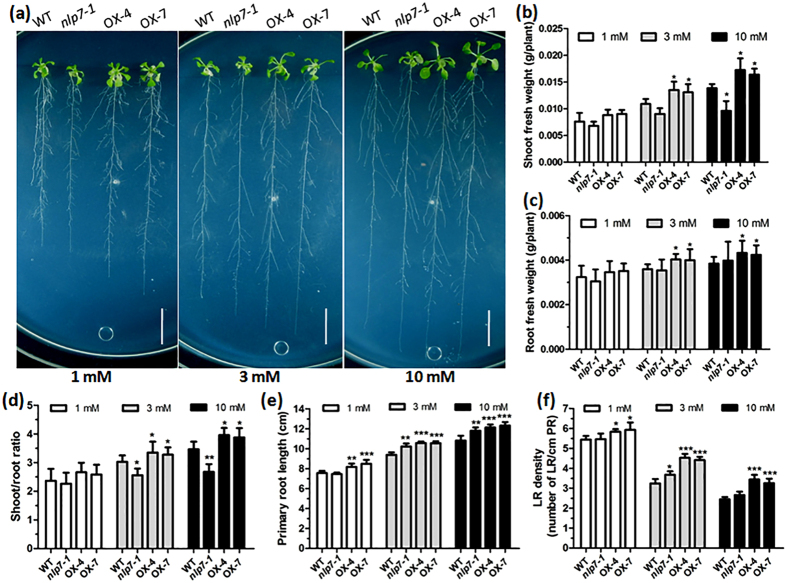
Root architecture and shoot/root ratio of *NLP7*-overexpressing, WT and *nlp7-1* plants. (**a**) The phenotypes of the 14-day-old plants on vertical plates containing different concentrations of nitrate. Diameter of the plate is 14.5 cm. Bar = 1.5 cm. (**b–f**) Shoot fresh weight (**b**), root fresh weight (**c**), shoot/root ratio (**d**), primary root length (**e**) and lateral root density (**f**) of the plants under different nitrate conditions. Values are the mean ± SD of six independent replications each containing 5 plants per genotype (*P < 0.05, **P < 0.01).

To verify the root architecture under different nitrate conditions, we checked the root system of the plants vertically grown on media containing different concentrations of nitrate. The results showed in Fig. 2a–c indicated that overexpression of *NLP7* conferred higher shoot and root FW compared with WT plants under both N-rich and -limiting conditions, though not so significantly under 1 mM nitrate condition. The *nlp7-1* plants showed reduced shoot FW compared with WT plants under different nitrate conditions, with more significantly reduced under higher nitrate conditions (Fig. 2b). However, there was no significant difference of root FW between the *nlp7-1* and WT plants. On the contrary, root FW of the *nlp7-1* mutant had slightly increased under 10 mM nitrate condition (Fig. 2c). Consequently, the shoot to FW weight ratio, an important parameter for nutrient starvation^31^, was significantly higher for *NLP7*-overexpressing plants and much lower for the *nlp7-1* plants under different nitrate conditions, especially under N-rich conditions (Fig. 2d). More detailed analysis of the root showed that the *NLP7*-overexpressing plants significantly increased PR length and lateral root (LR) density under both nitrate-rich and -poor conditions. However, the *nlp7-1* plants developed longer PRs and increased LR density under 3 mM and 10 mM nitrate conditions compared with WT plants (Fig. 2e,f). These results were confirmed by the data of time-course analysis of root development as shown in Supplementary Fig. S6, where *NLP7*-overexpressing plants had better-developed root system with increased LR number and slightly longer PRs compared with WT under both low and high nitrate conditions. However, the *nlp7-1* plants developed slightly more LRs and longer PRs compared with WT under 10 mM nitrate condition. In contrast, its LR number had a slight reduction with no obvious difference in PR length under 1 mM nitrate condition. The phenotypes of *nlp7-1* plants agree with the previous results that *NLP7* knockout confers constitutive N-starved phenotypes under different N conditions^29^. These data indicate that *NLP7* may be involved in root development and overexpression of *NLP7* promotes root development.

### Enhanced N uptake and assimilation in *NLP7*-overexpressing plants

**Figure 3:**
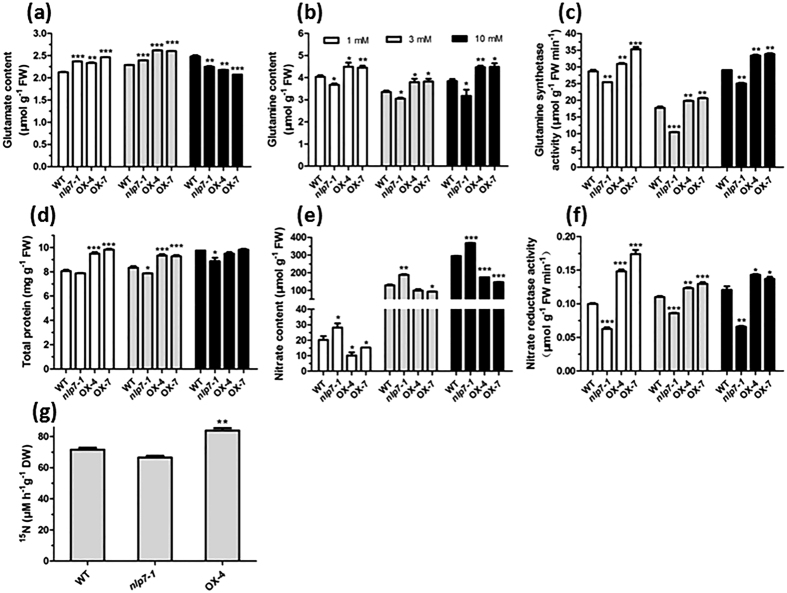
Enhanced N assimilation and nitrate uptake in *NLP7*-overexpressing plants. 16-day-old seedlings grown on agar medium with different concentrations of nitrate were used for metabolite analyses and enzymatic assays as described in Material and Methods. Values are the mean ± SD of three replications (*P < 0.05, **P < 0.01, ***P < 0.001). (**a,b**) Contents of glutamate (**a**), glutamine (**b**) in the plants grown under 1 mM, 3 mM and 10 mM nitrate conditions. (**c**) Enzyme activities of glutamine synthetase in the plants under different nitrate conditions. (**d,e**) Content of total protein (**d**) and nitrate (**e**) in the plants under different nitrate conditions. (**f**) Enzyme activities of nitrate reductase in the plants under different nitrate conditions. (**g**) Nitrate uptake activity assay. 10-day-old seedlings were labeled with 5 mM ^15^NO_3_^−^for 30 min and the amount of ^15^NO_3_^−^ taken into the plants was measured. Values are the mean ± SD of three replications (**P < 0.01). DW, dry weight.

To gain molecular insights for the improved growth of the *NLP7* transgenic plants under different N conditions, we measured several metabolite markers for N assimilation. Contents of glutamine (Gln) and glutamate (Glu), markers for N utilization, were measured respectively. Interestingly, we found that both the *NLP7*-overexpressing and mutant plants had higher Glu contents than WT plants under 1 mM and 3 mM nitrate conditions. Under 10 mM nitrate condition, Glu content increased in the WT plant, but decreased dramatically in *NLP7*-overexpressing plants (Fig. 3a). However, under this condition, Gln content was significantly higher in the *NLP7*-overexpressing plants, whereas was significantly lower in the *nlp7-1* mutant (Fig. 3b). These data implies that more Glu is converted to Gln in the *NLP7*-overexpressing plants than in the mutant plants under nitrate-rich conditions. Glu and Gln contents and their ratio are controlled by GS/GOGAT cycle, which is affected by many factors, such as GS/GOGAT enzyme activities, ammonium assimilation, 2-oxoglutarate (2OG) production and photorespiration^32^,^33^. In fact, we found the GS enzyme activities were much higher in the *NLP7*-overexpressing plants while significantly lower in the mutant (Fig. 3c), which might partly explain the Glu and Gln contents in the plants. Total protein content increased markedly in the *NLP7*-overexpressing plants under 1 mM and 3 mM nitrate conditions but not under 10 mM nitrate conditions compared with WT plants, while in *nlp7-1* mutant, the total protein content was significantly lower at 3 and 10 mM nitrate but not at 1 mM nitrate compared with the WT plants (Fig. 3d). Interestingly, we found that nitrate accumulated considerably in the *nlp7-1* mutant under different nitrate conditions, especially under high nitrate conditions. Conversely, nitrate content in the *NLP7*-overexpressing plants decreased significantly (Fig. 3e). These results led us to test the enzymatic activities of NR. Figure 3f shows that NR activities increased markedly in the *NLP7*-overexpressing plants in contrast to the dramatic decrease in the *nlp7-1* mutant, suggesting higher nitrate assimilation efficiency in the *NLP7*-overexpressing plants. Moreover, the *NLP7*-overexpressing plants exhibited a much higher nitrate uptake activity at 5 mM ^15^NO_3_^−^ external concentration (Fig. 3g).

### Overexpression of *NLP7* broadly up-regulates the expression of genes involved in N assimilation and signalling

**Figure 4:**
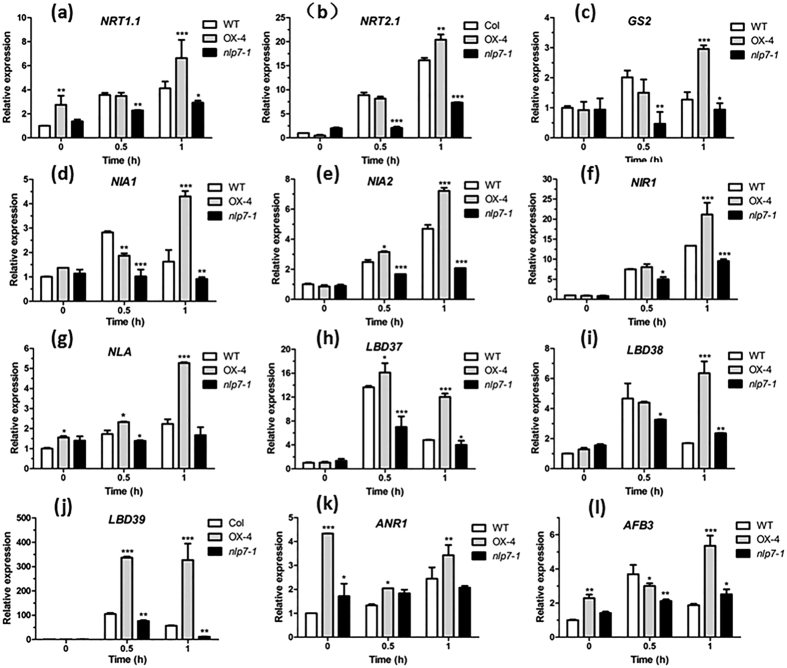
NLP7 broadly regulates the genes related to N utilization and signaling. 7-day-old plants grown on MS medium were transferred to N-free nutrient solution for 3 days, and then harvested for qRT-PCR analysis after re-supplied with 3 mM NO_3_^−^ for 0, 0.5 and 1 h. *UBQ5* was used as an internal control. NRT, nitrate transporter; GS2, glutamine synthetase 2; NIA, nitrate reductase; NIR1, nitrite reductase 1; NLA, nitrogen limitation adaptation; LBD, lateral organ boundary domain; AFB3, auxin signaling F-box 3. Values are the mean ± SD of three replications (*P < 0.05, **P < 0.01, ***P < 0.001).

To further assess the role of NLP7 in N assimilation in plant, we performed qRT-PCR to investigate the expression of some genes related with nitrate assimilation and signalling in 3 days N-starved seedlings and seedlings resupplied with nitrate for 0.5 h and 1 h, respectively. As showed in Fig. 4, after 3 days N-starved, most of the tested nitrate-responsive genes had similar expression levels in the WT, *nlp7-1* and *NLP7*-overexpressing plants, except *NRT1.1*, *NLA*, *ANR1* and *AFB3*, which had significant higher expression levels in the *NLP7*-overexpressing plants. However, after nitrate resupplied, almost all the genes were highly induced in WT and *NLP7*-overexpressing plants, but the induction levels were much lower in the *nlp7-1*. When resupplied with nitrate for 1 h, all these genes displayed considerable higher expression levels in the *NLP7*-overexpressing plants compared with in WT and *nlp7-1* plants. These results indicate that NLP7 plays a vital role in nitrate assimilation and signalling, which is consistent with the recent reports^28^. In addition, we checked the expression levels of all these genes in 7-day old plants grown on MS medium and observed that 11 of the 13 tested genes, except *NRT1.1* and *NIR1*, had much lower expression levels in the *nlp7-1* mutant. On the other hand, 10 out of the 13 genes showed prominent higher expression levels in the *NLP7*-overexpressing plants (Supplementary Fig. S7).

### NLP7 affects photosynthesis rate and C assimilation

**Figure 5:**
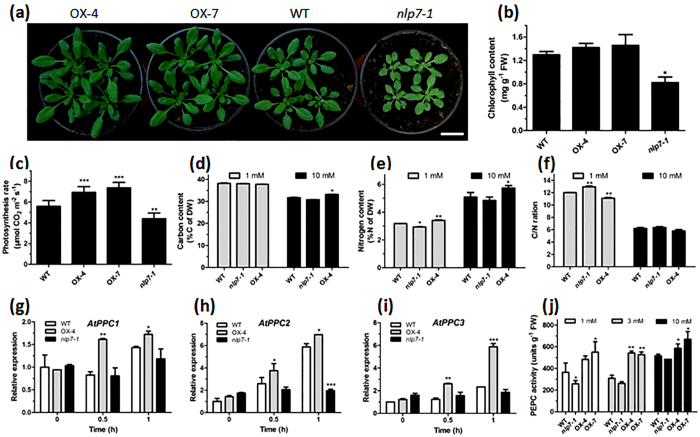
NLP7 enhances photosynthesis and affects C assimilation. (**a**) Image of 5-week-old *NLP7*-overexpressing, *nlp7-1* and WT plants grew in N rich soil. Bar = 2.5 cm. (**b**) Chlorophyll contents of the rosette leaves of the 5-week-old plants. Values are the mean ± SD of three replications (*P < 0.05). (**c**) Comparisons of photosynthesis rate in the 5-week-old *NLP7*-overexpressing, *nlp7-1* and WT plants. Photosynthesis rate was measured as described in Experimental procedures. Two measurements were made for each plant, and eight plants were used for each line. Values are the mean ± SD (**P < 0.01, ***P < 0.001). (**d–f**) C content (**d**), N content (**e**) and C/N ratio (**f**) of the *NLP7*-overexpressing, *nlp7-1* and WT plants. 16-day-old seedlings grown under different nitrate conditions were used for C and N content measurements using the NC analyzer. Values are the mean ± SD of three replications (*P < 0.05, **P < 0.01). (**g–i**) Expression levels of the PEPC genes *AtPPC1* (AT1G53310) (**g**), *AtPPC2* (AT2G42600) (**h**), *AtPPC3* (At3G14940) (**i**). 7-day-old plants grown on MS medium were transferred to N-free nutrient solution for 3 days, and then harvested for qRT-PCR analysis after re-supplied with 3 mM NO_3_^−^ for 0, 0.5 and 1 h. *UBQ5* was used as an internal control. Values are the mean ± SD of three replications (*P < 0.05, **P < 0.01, ***P < 0.001). (**j**) PEPC activities in the plants grown under 1 mM, 3 mM and 10 mM nitrate conditions. 16-day-old seedlings grown under different nitrate conditions were used for enzymatic assays. Values are the mean ± SD of three replications (*P < 0.05, **P < 0.01).

N metabolism is known to coordinate with photosynthesis and C metabolism^34^. In this study, we found *nlp7-1* plants displayed N-starved phenotypes with a much smaller and pale rosette when grown in soil (Fig. 5a). Further analysis found chlorophyll content was remarkably reduced in the leaves of *nlp7-1* plants, whereas increased in the *NLP7*-overexpressing plants (Fig. 5b). Photosynthesis rate was enhanced in the *NLP7*-overexpressing plants and decreased in the *nlp7-1* plants (Fig. 5c). Furthermore, total C content displayed no significant difference between *NLP7*-overexpressing, *nlp7-1* and WT plants under 1 mM nitrate condition. However, compared with WT, total C content reduced by 2.8% in *nlp7-1*, while significantly increased by 4.7% in *NLP7*-overexpressing plants under 10 mM nitrate condition (Fig. 5d). Total N content was found decreased in *nlp7-1* while markedly increased in the *NLP7*-overexpressing plants compared with WT under both nitrate -rich and -deficient conditions (Fig. 5e). As a result, C/N ratio decreased significantly in the *NLP7*-overexpressing plants under low nitrate conditions, but not so significantly changed under high nitrate conditions compared with the WT plants (Fig. 5f).

We also measured some C metabolism-related compounds. Table 1 showed that there were more sucrose, fructose, and glucose accumulation in *NLP7* transgenic plants under 10 mM nitrate condition, and less sucrose and glucose under 1 mM nitrate condition. Conversely, *nlp7-1* plants accumulated more soluble sugars under 1 mM nitrate condition and less sucrose and fructose under 10 mM nitrate condition. Moreover, starch content, which is known to correlate negatively with biomass^35^, was found much lower in the *NLP7*-overexpressing plants under 1 mM nitrate condition. These data suggest that NLP7 may affect C metabolism.

In addition, we found that NLP7 affected the expression level of *AtPPC* genes, which encode the PEPC. *NLP7* transgenic plants showed higher transcript levels of *AtPPC1* in response to nitrate re-addition (Fig. 5g). Expression of *AtPPC2* and *AtPPC3* were induced by nitrate in both the WT and *NLP7*-overexpressing plants, but more dramatically in the *NLP7*-overexpressing plants. However, expression of these two genes did not change much in the *nlp7-1* mutant after nitrate re-addition (Fig. 5h,i). These data indicate NLP7 may affect PEPC activities. Indeed, further analyses showed that PEPC activities increased noticeably in the *NLP7*-overexpressing plants while reduced in the *nlp7-1* plants, especially under N-limiting conditions (Fig. 5j). Meanwhile, *NLP7*-overexpressing plants had higher expression levels of genes encoding cytosolic isocitrate dehydrogenase (ICDH), mitochondrial ICDH and peroxisomal ICDH after 3 days N-starvation and 0.5 h of N re-addition, with only increased expression of cytosolic ICDH gene after 1 h of nitrate re-addition (Supplementary Fig. S8). These results suggest that *NLP7* expression level influenced not only N assimilation but also C assimilation.

### NLP7 enhanced N assimilation and growth of the transgenic tobacco

**Figure 6:**
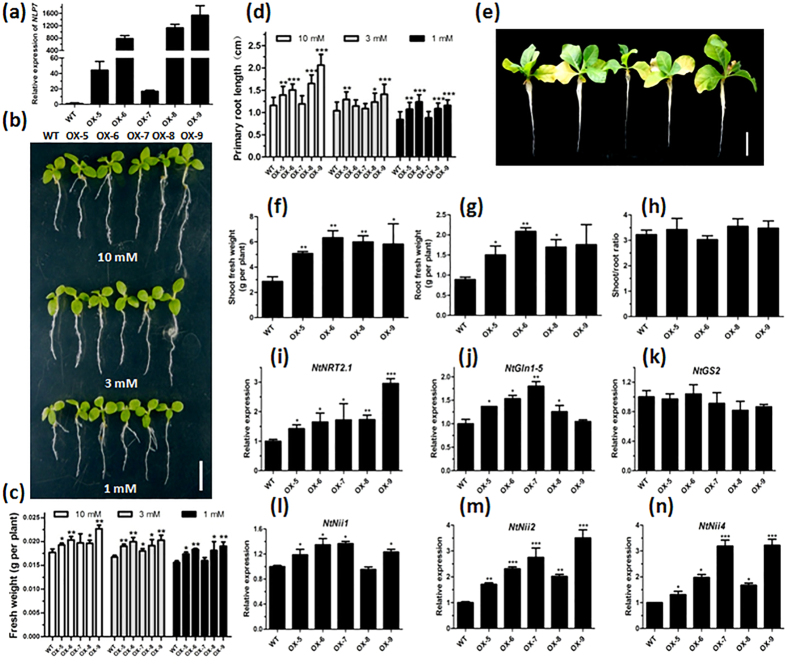
NLP7 enhances N assimilation and growth of transgenic tobacco plants. (**a**) Identification of the *NLP7* transgenic tobacco by qRT-PCR. 15-day-old seedlings grown on medium containing 10 mM nitrate were used for RNA extraction and qRT-PCR. Ubiquitin-conjugating enzyme E2 (*NtUbc2*, accession number AB026056) was used as an internal control^62^. Values are the mean ± SD of three replications. (**b**) Images of the 15-day-old WT and *NLP7* transgenic tobacco plants grown on medium with different concentrations of nitrate. Bar = 0.5 cm. (**c,d**) FW (**c**) and PR length (**d**) of the 15-day-old WT and transgenic tobacco plants. Values are the mean ± SD of three replications each containing 10 plants per genotype (*P < 0.05, **P < 0.01, ***P < 0.001). (**e–h**) Tobacco plants grown hydroponically under 1 mM nitrate condition (**e**), shoot FW (**f**), root FW (**g**) and shoot/root FW ratio (**h**) of the tobacco plants. 20-day-old tobacco seedlings grown on MS medium were used for hydroponic culture for 28 days. Values are the mean ± SD of 6 plants (*P < 0.05, **P < 0.01). Bar = 7 cm. (**i–n**) NLP7 up-regulated the expression levels of N assimilation related genes in tobacco. 15-day-old seedlings grown on medium containing 10 mM nitrate were used for RNA extraction and qRT-PCR. Expression levels of six tobacco genes were quantified by qRT-PCR: nitrate transporter (*NtNRT2.1*, accession number AJ557583), cytosolic glutamine synthetase (*NtGln1-5*, accession number X95932), plastidic glutamine synthetase (*NtGS2*, accession number X95932S39536), nitrite reductase (*NtNii1*, accession number X66145; *NtNii2*, accession number AB103507; *NtNii4*, accession number AB093534). *NtUbc2* was used as an internal control. Values are the mean ± SD of three replications (*P < 0.05, **P < 0.01, ***P < 0.001).

In order to investigate whether NLP7 can similarly modulate N assimilation in different plant species, we generated *NLP7*-ovexpressing transgenic tobacco plants. Figure 6a–c showed that the transgenic tobacco plants exhibited growth advantages with increased FW compared with WT under different nitrate conditions. The *NLP7*-overexpressing tobacco plants also had much longer PRs (Fig. 6d). In addition, we assayed the N use level of the transgenic tobacco using hydroponic culture method. The results showed that the *NLP7*-overexpressing plants grew much better with higher shoot and root biomass under 1 mM nitrate condition (Fig. 6e–g). The shoot/root ratios were increased in three of the four *NLP7* overexpression lines (Fig. 6h). Moreover, compared with WT, five of the six tested genes in the N assimilation pathway showed significant higher transcript levels in the transgenic plants compared with the WT plants (Fig. 6i–n). These results suggest that the strategy with NLP7 may be applicable to improve N use in other plant species.

## Discussion

Decreasing fertilizer N inputs by improving plant NUE is an important strategic goal for global agriculture. However, traditional breeding strategies to improve NUE in some crop plants have experienced a plateau^6^. Although genetic engineering for improving N use has been broadly studied in recent decades, the success has been limited because of focusing on single gene manipulation. Most of these studies attempted to improve plant N use by individually constitutive overexpression of the genes involved in N uptake or metabolism^2^. Unfortunately, most of these transgenic plants did not show increased NUE, and some even showed negative pleiotropic effects, indicating the notions of single-point rate-limiting regulation being oversimplified^5^,^6^,^36^. Probably, the effects of alterations in the amount and/or activity of any single enzyme in the N metabolic or uptake pathway may be masked by concurrent mechanisms that are activated to maintain homoeostasis, including post-transcriptional, translational and ⁄or feedback regulation^3^,^22^. In addition, unnecessary accumulation of metabolic intermediates may affect the plant development. Therefore, it should be much better to engineer the plant metabolism by enhancing a few steps cooperatively instead of one step in a metabolic pathway to avoid these weaknesses. The introduction of the *ZmDof1* gene into *Arabidopsis* and rice highlighted the great utility of TFs in engineering N and C metabolisms in plants^21^,^22^. In this study, we found another TF, NLP7, could simultaneously coordinate many processes in N utilization and signaling pathway as well as C fixation and metabolism pathway, resulting in improved N use and plant growth.

Recently, NLPs had proved to play a central role in nitrate signaling in *Arabidopsis.* One mechanism of *NLPs* modulates nitrate-induced gene expression probably through post-translational regulation^27^. The underlying mechanisms for how inactive form of NLPs been converted into active form by nitrate signalling are not clear. However, according to the ATH1 chip data by Scheible *et al*.^20^, 7 *NLP* genes of the N-depleted seedlings responded quickly and transiently to nitrate re-addition, with *NLP3* being the most responsive. Based on data from GENEVESTIGATOR database, Chardin *et al*.^37^ found the expression of *NLP8* responds to a large number of treatments, including several N nutrient treatments, whereas *NLP4* and *NLP9* are responsive to specific stimuli only, such as heat stress and N treatments, respectively. Transcription of *OsNLP4* is repressed by several abiotic stress treatments and induced by low phosphate availability^37^. Konishi and Yanagisawa^27^ found all the *NLPs* were not significantly induced by nitrate re-addition for one hour. However, this result can not eliminate the possibility that the expression of different *NLPs* being regulated by nitrate at other time points. Moreover, constitutive overexpression of *NLP6-VP16* using the 35S promoter up-regulated the expression of nitrate-inducible genes *NIR1* and *NIA2* in both the N-starved seedlings and seedlings resupplied with nitrate^27^. All these results imply that not only post-translational but also transcriptional regulation of NLPs probably plays roles in the regulation of N signalling and assimilation. Based on this hypothesis, it is possible for us to improve plant N use by change the transcriptional levels of NLPs.

NLP7 was reported to orchestrate the early response to nitrate^28^,^29^. However, the expression of *NLP7* is not induced by the N source or nitrate^29^, but the localization of NLP7 protein is regulated by nitrate via a nuclear retention mechanism^28^. Therefore, it is reasonable to speculate that if we overexpressed *NLP7* in plant, more NLP7 protein would be produced and accumulated in the nucleus in response to nitrate availability. This hypothesis could be proved by the results of Marchive *et al*.^28^ (see supplementary Fig. S4 of the paper). In this figure, less NLP7-GFP protein was accumulated in both the cytosol and the nucleus of root cells of N-starved pNLP7:NLP7-GFP plantlets compared with that of N-starved p35S:NLP7-GFP plantlets. Once nitrate was re-supplied, within minutes, noticeably more NLP7-GFP protein was accumulated in the nucleus of root cells in p35S:NLP7-GFP plants compared with that of pNLP7:NLP7-GFP plantlets. As a result, the more NLP7 protein accumulated in nuclear would enhance the expression of N assimilation genes and thus improve the N use ability of the *NLP7*-overexpressing plants.

Indeed, our results support this hypothesis that the overexpression of *NLP7* significantly enhanced the plant N use by enhancing N assimilation efficiency. Overexpression of *NLP7* in *Arabidopsis* led to higher shoot and root biomass under both N-rich and -deficient conditions (Figs 1 and 2, Supplementary Figs S3 and S5), with remarkable rise in multiple N metabolites and N content, an elevation in enzyme activities of N metabolism (Figs 3 and 5). On the contrary, the *nlp7-1* mutant had impaired N use ability and showed more severe N-deficient phenotypes, even under N rich conditions (Figs 1 and 2). In sharp contrast to the *nlp7-1*, overexpression of *NLP7* resulted in up-regulation of a range of genes involved in nitrate transport (*NRT1.1*, *NRT2.1*), N assimilation (*GS1*, *NIA1*, *NIA2*, *NIR1*), and N signalling (*LBD37*, *LBD38*, LBD39, *ANR1*, *AFB3*) (Fig. 4, Supplementary Fig. S7). Increased transcript levels of these genes would lead to enhanced N uptake and metabolism as revealed in Fig. 3. On the other hand, the up-regulated TFs would produce a broad range of regulatory outcomes. These coordinated regulations by NLP7 enable plants to rapidly adapt to N availability and maintain plant N homeostasis. Notably, transcript level of *NLA*, a positive regulator of plant adaptation to N limitation^38^, was also found up-regulated in *NLP7*-overexpressing plants (Fig. 4h, Supplementary Fig. S7), contributing to better performances of the transgenic plants under N-deficient conditions. Moreover, ectopic expression of *Arabidopsis NLP7* in tobacco also had similar effects as in *Arabidopsis* (Fig. 6), suggesting that this might be a conserved mechanism in plants. All these data indicate that overexpression of *NLP7* can improve plant N use ability by coordinately regulating N metabolism, transport and signalling pathways.

Root is the most important organ for sensing N availability and morphological adaptation to N supply^39^. Agreed with the previous study^29^, our results also found that *NLP7* was highly expressed in leaves, central cylinder of roots and LR primordia at different stages (Supplementary Fig. S9), implying a possible function of this gene in root development. In fact, our results revealed that overexpression of *NLP7* conferred increased LR density and PR length under both high and low nitrate conditions by vertical growth assay (Fig. 2 and Supplementary Fig. S6). The expression of many genes involved in root development in response to N availability, such as *NRT1.1*, *NRT2.1*, *ANR1* and *AFB3*^40^,^41^,^42^,^43^, changed significantly in the *NLP7*-overexpressing and knockout plants (Fig. 4). The up-regulation of these genes in the *NLP7*-overexpressing plants probably contributes to the root architecture changes to some extent. Overall, these morphological changes in the root system of *NLP7*-overexpressing plant enhanced its N acquisition ability to match its high-efficient N metabolism.

Plants have the ability to optimize biomass partitioning to maximize whole-plant growth rate according to the external environment^44^. According to Thornley’s model, under N deficiency stress, the greater part of the N taken up was used for root growth, thus decreasing shoot/root ratio^45^,^46^. Therefore, shoot/root ratio can reflect the N use level and adaptation ability to N deficiency. Our results showed that the *nlp7-1* displayed typical N-starved phenotypes with decreased shoot/root ratios, irrespective of N supply. However, the *NLP7*-overexpressing plants had much higher shoot/root ratios, particularly under nitrate-rich conditions (Figs 2d and 6h). These results implied that *NLP7*-overexpressing plant has higher N use ability, thus leading to more tolerance to the N deficiency and more N allocated to the shoot to maximize its relative growth rate.

N and C assimilation processes are closely linked and tightly co-regulated^34^. NUE is not only dependent on N assimilation, and manipulating C metabolism was useful in some cases in improving NUE^47^. Therefore, it is reasonable to improve both the C and N utilization efficiencies simultaneously to optimize plant growth and yield. Interestingly, we found such cooperation between N and C assimilations in the *NLP7*-overexpressing plants. In addition to the increased N content (Fig. 5e), *NLP7* -overexpressing plants also had significantly increased C content compared with that of the *nlp7-1* mutant under N-rich condition (Fig. 5d). Overexpression of *NLP7* conferred significant higher photosynthesis rate (Fig. 5c). The changed chlorophyll contents of the *NLP7*-overexpressing and mutant plants (Figs 1c,d and 5b) may have direct influences on C fixation (Fig. 5c). Moreover, expression levels of PEPC genes and ICDH genes were found up-regulated in the *NLP7*-overexpressing plants after nitrate induction (Fig. 5g–i, Supplementary Fig. S8). Consequently, the *NLP7*-overexpressing plants had much higher PEPC activities under both N-rich and -deficient conditions (Fig. 5j). According to the ChIP-chip data by Marchive *et al*.^28^, one of our tested PEPC genes (*AtPPC2*) and *ICDH* genes (cytosolic ICDH) are bound by NLP7 in the presence of nitrate (see Supplementary Data 1 and 8 of the paper), suggesting that NLP7 may directly regulate the transcription of these PEPC and ICDH genes. It has been well documented that PEPC, a key enzyme in photosynthesis, also acts as a key player in N storage and C fixation and as a crosstalk point between C and N metabolisms^21^,^48^. ICDH genes encode the key enzymes to provide 2OG necessary for ammonium assimilation^49^. Therefore, up-regulation of these genes might positively regulate C assimilation as well as N assimilation, thus affecting the C/N balance under N-rich conditions (Fig. 5f). Balanced C and N nutrient provisions are beneficial to ensure maximal N use and to maintain an appropriate shoot/root ratio for plant development and growth.

In conclusion, we have demonstrated that *NLP7* is potentially a promising candidate for improving plant N use ability. The localization of NLP7 is controlled by nitrate via a nuclear retention mechanism^28^. Amount of NLP7 protein in the cytosol and nucleus may maintain a dynamic balance in response to the nitrate availability. Constitutively overexpression of *NLP7* in the plant might break the normal balance of NLP7 localization between the cytosol and nucleus and promoted NLP7 protein relocation to the nucleus, especially under N-rich condition. Being activated by nitrate signalling^27^, the nuclear accumulated NLP7 would enhance N assimilation by cooperatively modulating a number of genes related to N metabolism, transport and signalling. Consequently, the overexpression of *NLP7* conferred better growth under both N-deficient and -sufficient conditions. Moreover, overexpression of *NLP7* also improved C assimilation simultaneously. Our results also imply that *NLP7*-mediated nitrate regulation is not only through post-translational mechanisms, probably also through translational levels. It is conceivable that *NLP7* can be used to enhance N use ability and increase crop yield.

## Material and Methods

### Plant material

The *nlp7-1* (SALK_26134C) mutant was obtained from Arabidopsis Biological Resource Center (ABRC). Homozygous mutant plants were confirmed by RT-PCR using the primers *NLP7* RT-PCR LP and RP. 35S:*NLP7* overexpression construct was made by inserting the coding region of *NLP7* amplified by PCR using *NLP7*-attb-LP and *NLP7*-attb-RP into pCB2004^50^ via GATEWAY cloning system. For pNLP7:NLP7–GFP construct, a fragment containing *NLP7* promoter and coding region amplified by genomic PCR with primers *NLP7*-attb-LP and *NLP7*-attb-RP2 was cloned into pMDC110 to fuse with GFP^51^. The *NLP7*-overexpressing transgenic *Arabidopsis* were obtained by *Agrobacterium*-mediated floral-dip method^52^ and identified by qRT-PCR with specific primers *NLP7*-qPCR LP and RP. The transgenic tobacco plants were generated as previously described^53^ and identified by RT-PCR with the primers *NLP7* RT-PCR LP and RP. All the primers used are listed in Table S1.

### Plant growth conditions

Seeds were sterilized with 15% bleach for 12 min, and then washed five times with sterile water. Sterilized seeds stratified at 4 °C for 2 days, and plated on solid medium containing 1% (w/v) sucrose and 0.6% (w/v) agar. Nitrate-less medium was modified on MS medium with KNO_3_ as sole N source: 10 mM nitrate medium (similar to MS except 20 mM KNO_3_ and 20 mM NH_4_NO_3_ was replaced with 10 mM KCl and 10 mM KNO_3_), 3 mM nitrate medium (similar to MS except 20 mM KNO_3_ and 20 mM NH_4_NO_3_ was replaced with 7 mM KCl and 3 mM KNO_3_), 1 mM nitrate medium (similar to MS except 20 mM KNO_3_ and 20 mM NH_4_NO_3_ was replaced with 9 mM KCl and 1 mM KNO_3_).

To investigate different growth rates under different N conditions, seeds were germination and grew on medium containing different concentrations of nitrate at 22 °C under 16-h light/8-h dark photoperiod. For evaluation the phenotype of soil-grown plants, seeds were germinated and grew in N-limited soil without N fertilizer applying during the whole growth stage at 22 °C under 10-h light/14-h dark photoperiod. N-limited soil was the soil had been used once for *Arabidopsis* growth.

### RNA extraction and qRT-PCR

1 μg of total RNA isolated using Trizol reagent (Invitrogen, Carlsbad, California, USA) were used for reverse transcription. qRT-PCR was performed with a StepOne Plus Real Time PCR System by using a TaKaRa SYBR Premix Ex Taq II reagent kit. All the primers used are shown in Table S1.

### Metabolite analyses

The metabolite analyses were performed on the seedlings of 16-day-old plants grown on agar medium with different concentrations of nitrate. Total soluble protein was measured using the Bradford Protein Assay Kit (Sangon Biotech, Shanghai, China) and total amino acid according to Rosen^54^. Concentrations of glutamine and glutamate were determined with the glutamine/glutamate determination kit (GLN-1; Sigma-Aldrich). Sucrose, fructose, and glucose content were measured using assay kits (Su Zhou Keming Bioengineer Company, China) following to the manufacturer’s instructions. Then starch was extracted by incubating the pellets with 35% HClO_4_ overnight and the glucose liberated was analyzed by anthrone-H_2_SO_4_ method^55^. Chlorophyll a and b were extracted entirely from the aerial parts by using 80% acetone, and measured by spectrophotometric method as Arnon^56^. Nitrate was extracted in 50 mM HEPES–KOH (pH 7.4), and measured by the method according to Cataldo *et al*.^57^. The percent total N and C content in oven-dried plant material was measured with an NC analyzer (Vario EL III model, Elementar, Hanau, Germany) according to the manufacturer’s instructions.

### Enzyme activity assays

Enzymes were extracted from 16-day-old plants grown on agar medium with different concentrations of nitrate. The maximum *in vitro* activities of NR was assayed as described previously^58^. GS enzyme activities was measured according to Cai *et al*.^59^. PEPC activities were measured by an enzyme-coupled spectrophotometric method using assay kit (Su Zhou Keming Bioengineer Company, China) following to the manufacturer’s instructions.

### Photosynthesis rate measurement

Photosynthesis rates were measured using a portable photosynthesis system (LI-COR LI-6400XT) in the morning (9:30 to 11:30 AM) under constant light in the greenhouse as described by Yu *et al*.^60^. All of the measurements were taken at a constant air flow rate of 500 μmol s^−1^. The CO_2_ concentration was set as 400 μmol mol^−1^ using the system’s CO_2_ injector (model 6400-01, Li-COR).

### Hydroponic culture

Tobacco seeds were sterilized with 15% bleach for 15 min and germination on MS agar medium at 22 °C under 16-h light/8-h dark photoperiod. 20-day-old seedlings were used for hydroponic culture. The roots of the tobacco seedling were wrapped with sponge and then grown on a support made of thick polystyrene foam board with holes to allow the root systems of the plants to grow into the hydroponic solution. The floating body floated on hydroponic solution with 1 mM nitrate (similar to MS nutrient solution except 20 mM KNO_3_ and 20 mM NH_4_NO_3_ was replaced with 9 mM KCl and 1 mM KNO_3_). Plants were cultured at 22 °C under 16-h light/8-h dark photoperiod. Nutrient solution was changed every 5 days.

### Nitrate uptake assay using ^15^NO_3_^−^

The nitrate uptake activity was assayed using ^15^NO_3_^−^ as described previously^61^. 10-day-old seedlings were transferred first to 0.1 mM CaSO_4_ for 1 min, then to modified MS nutrient solution with 5 mM K^15^NO_3_^−^(99% atom) as sole N source for 30 min and finally to 0.1 mM CaSO_4_ for 1 min. Seedlings were then dried at 70 °C to a constant weight and grinded. ^15^N content was analyzed using a continuous-flow isotope ratio mass spectrometer (Thermo-MAT253) coupled with a elemental analyzer (Flash 2000 HT, Thermo Fisher Scientific, Inc., USA).

### Statistical analysis

Statistically significant differences were computed based on the Student’s *t*-tests.

## Additional Information

**How to cite this article**: Yu, L.-H. *et al*. Overexpression of *Arabidopsis NLP7* improves plant growth under both nitrogen-limiting and -sufficient conditions by enhancing nitrogen and carbon assimilation. *Sci. Rep.* **6**, 27795; doi: 10.1038/srep27795 (2016).

## Supplementary Material

Supplementary Information

## Figures and Tables

**Table 1:** Carbohydrate contents of 16-day-old WT, *nlp7-1* mutant and *NLP7*-overexpressing plants grown under different nitrate conditions.

Nutrient medium	Line	Carbohydrate content (μmol g^−1^ FW)			
		Sucrose	Fructose	Glucose	Starch
10 mM	WT	16.13 ± 2.85	5.46 ± 0.61	2.03 ± 0.11	6.12 ± 0.20
	*nlp7-1*	12.31 ± 2.61	4.89 ± 0.63	3.55 ± 0.05^***^	5.89 ± 0.23
	OX-4	25.19 ± 3.85^*^	9.56 ± 0.30^**^	2.99 ± 0.09^***^	6.31 ± 0.26
	OX-7	24.28 ± 3.43^*^	7.46 ± 0.01^*^	3.24 ± 0.10^***^	5.87 ± 0.07
1 mM	WT	28.12 ± 4.05	10.97 ± 0.63	13.19 ± 0.09	47.14 ± 0.09
	*nlp7-1*	33.12 ± 1.42	14.53 ± 0.80^*^	16.12 ± 0.10^***^	49.01 ± 0.30
	OX-4	20.39 ± 2.14^*^	12.98 ± 0.53^*^	9.87 ± 0.16^***^	38.96 ± 0.30^**^
	OX-7	19.05 ± 0.90^*^	12.22 ± 0.36	11.16 ± 0.24^***^	41.89 ± 1.01^*^

Data are means ± SD of three independent replications (^*^P < 0.05, ^*^^*^P < 0.01, ^*^^*^^*^P < 0.001).
