# Supplementary material for: Overexpression of Arabidopsis NLP7 improves plant growth under both nitrogen-limiting and -sufficient conditions by enhancing nitrogen and carbon assimilation
Source: Sci Rep. 2016 Jun 13;6:27795. doi: 10.1038/srep27795 (PMC4904239; doi:10.1038/srep27795)
Supplement: Supplementary Information [file srep27795-s1.doc]

**Supplementary Information**

**Overexpression of *Arabidopsis* *NLP7* improves plant growth under both nitrogen-limiting and -sufficient conditions by enhancing nitrogen and carbon assimilation**

Lin-Hui Yu^1^†, Jie Wu^1^†, Hui Tang^1^, Yang Yuan^1^, Shi-Mei Wang^2^, Yu-Ping Wang^3^, Qi-Sheng Zhu^2^, Shi-Gui Li^3^ & Cheng-Bin Xiang^1*^

Figure S1. Identification of *NLP7* mutant and overexpression lines.

(a) Schematic diagram of the T-DNA region of expression vector pCB2004-*NLP7.* In order to efficiently express the *NLP7* in *Arabidopsis*, *NLP7* cDNA was constructed into the expression binary vector pCB2004. *LB* left border, *RB* right border, *35S polyA* Cauliflower mosaic virus (CaMV) 35S polyA, *bar* phosphinotricin acetyltransferase gene, *35S promoter* CaMV 35S promoter, *NOS polyA* nopaline synthetase gene polyA.

(b) *NLP7* gene structure schematic model with the sites of T-DNA insertion. Squares correspond to exons while lines represent introns.

(c) *NLP7* transcript level in the transgenic lines and mutants, as revealed by qRT-PCR analysis. Only 2.6% of the wild-type *NLP7* mRNA level was found in *nlp7-1* mutant, while the three transgenic lines had obvious overexpression of *NLP7.* Values are the mean ± SD of three independent repetitions. *UBQ5* was used as the internal control.

(a)

(b)

(c)

Figure S2. Analysis of expression levels of *NLPs* in *NLP7*-overexpressing plants, WT and *nlp7-1* plants by qRT-PCR. *UBQ5* was used as an internal control. Values are the mean ± SD of three independent repetitions.

Figure S3. The phenotypes of 18-days-old WT, *nlp7-1* and *NLP7*-overexpressing plants vertically grown on medium with different concentrations of nitrate. Seeds were germinated and grew vertically on medium containing 1 mM, 3 mM, 10 mM nitrate for 18 days. Diameter of the plate is 14.5 cm. Bar = 1 cm.

Figure S4. Complementation of *Arabidopsis* *nlp7-1* mutant.

pNLP7:NLP7–GFP construct was introduced into the *Arabidopsis* *nlp7-1* mutant. Two transgenic homozygotes (4-2 and 9-6) were used for functional complementation analysis. Seeds were geminated on medium with 10 mM or 1 mM nitrate for 7 days.

(a) Expression analysis of *NLP7* in 7-d-old WT, pNLP7:NLP7–GFP transgenic homozygotes and *nlp7-1* plants grown on medium with 10mM nitrate by qRT-PCR.

(b) Phenotypes of 7-d-old WT, pNLP7:NLP7–GFP transgenic homozygotes and *nlp7-1* plants grown on medium with 10 mM and 1 mM nitrate. Bar = 0.5 cm.

(c-d) FW (c) and primary root length (d) of the 7-d-old WT, pNLP7:NLP7–GFP transgenic homozygotes and *nlp7-1* plants.

Figure S5. Phenotypes of WT, *nlp7-1* and *NLP7*-overexpressing plants suffering N-starvation and grown in N-limiting soil.

(a) The phenotypes of WT, *nlp7-1* and *NLP7*-overexpressing plants after 3 days N starvation. 7-day-old plants grown on MS medium were transferred to N-free sterile nutrient solution and grew on orbital shakers (60 rpm) for 3 days. Bar = 1.5 cm.

(b-c) Photographs of the 7-week-old plants grown in N-limiting soil. The plants were grown on agar plates for one week and further grown in soil for 6 weeks under 10-h light/14-h dark photoperiod. Bar in figure a is 5 cm, in figure b is 2 cm.

(d-f) Shoot fresh weight (d), root fresh weight (e) and primary root length (f) of the 7-week-old plants grown in soil. Values are the mean ± SD of three independent repetitions of 6 plants each. Student’s *t*-test, *P < 0.05, **P < 0.01.

Figure S6. Root architecture of WT, *nlp7-1* and *NLP7-*overexpressing plants grown on vertical plate containing 1 mM and 10 mM nitrate.

The seeds were germinated and growth on vertical plates. Visible lateral root number was counted every day from the 6th day, and the primary root tips were marked. On the 11th day, pictures of the plates were taken, and digital images of plants were used for root length measurement by hand using ImageJ software (NIH).

(a-b) Lateral root (a) and primary root (b) growth curves of WT, *nlp7-1* and *NLP7-*overexpressing plants under 1 mM nitrate conditions. Values are the mean ± SD of three independent experiments each containing 15-20 plants per genotype.

(c-d) Lateral root (c) and primary root (d) growth curves of WT, *nlp7-1* and *NLP7-*overexpressing plants under 10 mM nitrate conditions. Values are the mean ± SD of three independent experiments each containing 15-20 plants per genotype.

Figure S7. Expression levels of the genes related to N utilization and signaling in the 7-day-old plants grown on MS medium.

7-day-old seedlings grown on MS medium were used for RNA extraction and qRT-PCR analyses. *UBQ5* was used as an internal control. Values are the mean ± SD of three independent repetitions. Student’s *t*-test, *P < 0.05, **P < 0.01, ***P < 0.01.


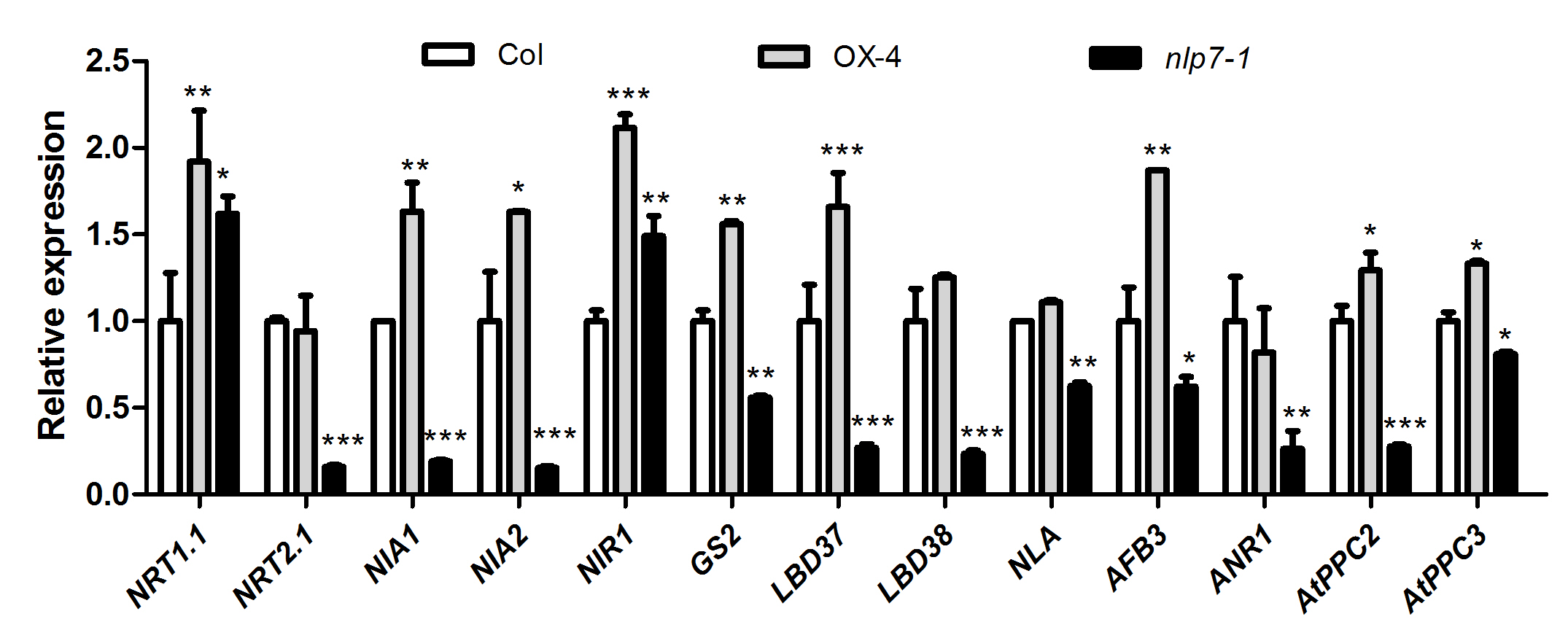


Figure S8. NLP7 affects the expression levels of ICDH genes.

7-day-old plants grown on MS medium were transferred to N-free nutrient solution for 3 days, and then harvested for qRT-PCR analysis after re-supplied with 3 mM NO_3_^-^ for 0, 30 and 60 min. *UBQ5* was used as an internal control. cytosolic ICDH (AT1G65930), peroxisomal ICDH (AT1G54340), mitochondrial ICDH (AT5G14590).Values are the mean ± SD of three independent repetitions. Student’s *t*-test, *P < 0.05, **P < 0.01, ***P < 0.01.

Figure S9. Expression pattern of *NLP7* revealed by GUS staining of *pNLP7::GUS* transgenic seedlings. GUS activity was observed in seedling of 2-day-old (a), seedling of 4-day-old (b), seedling of 7-day-old (c), seedling of 9-day-old (d), seedling of 14-day-old (e), seedling of 20-day-old (f), lateral root primordia at different stages (g-k), lateral root (l).

Table S1. Primers used for PCR.

| Name | Sequence (5' to 3') |
| --- | --- |
| *NLP7* qRT-PCR LP | GAGTTTGCCCGACGACAATGAAG |
| *NLP7* qRT-PCR RP | GGCCTCCATCAGTACCTTGAACAG |
| *NRT1.1* qRT-PCR LP | GCACATTGGCATTAGGCTTT |
| *NRT1.1* qRT-PCR RP | CTCAATCCCCACCTCAGCTA |
| *NRT2.2* qRT-PCR LP | AGTCGCTTGCACGTTACCTG |
| *NRT2.2* qRT-PCR RP | AATGTCATGTTTGGTGAGGTTAAGA |
| *NIA2* qRT-PCR LP | AGGATCCAGAGGATGAGACTGAAA |
| *NIA2* qRT-PCR RP | CCTTAGCTGATTCCACTACGTACCA |
| *GS2* qRT-PCR LP | CACCAAACCTTACTCTCTGACA |
| *GS2* qRT-PCR RP | CACTATCTTCACCAGGTGCTTG |
| *NIR1* qRT-PCR LP | CCGGTAGCCAGTTCTGCG |
| *NIR1* qRT-PCR RP | CCTATTCGTCCCCCGACGT |
| *LBD37* qRT-PCR LP | TGCTTTGTTTCAGTCGTGCT |
| *LBD37* qRT-PCR RP | TGCTCCGTTAACTGGATTGACA |
| *LBD38* qRT-PCR LP | TGCCCTGCTTTGTTTCAGTCTT |
| *LBD38* qRT-PCR RP | CGTTCACCGGATTCACAGTTCT |
| *LBD39* qRT-PCR LP | GAACTCCAACGTCCTGCTTTGT |
| *LBD39* qRT-PCR RP | ATACCAACCGCTCCGTTAACC |
| *ANR1* qRT-PCR LP | TTGGCAAAGAGAGGTTGCAAG |
| *ANR1* qRT-PCR RP | TCTCCCACTAGTTTCCTGTGGC |
| *AFB3* qRT-PCR LP | TTCTTGCTGACGTGGGTAGGTAC |
| *AFB3* qRT-PCR RP | CTTCTCTCTTTCATCTTCTTCATTC |
| *NLA* qRT-PCR LP | GAGCGACTCTGTTTTGTTGATC |
| *NLA* qRT-PCR RP | CATGTACTCTTCATACTTCTTACA |
| AT1G53310 (*AtPPC1*) qRT-PCR LP | TGCTGCTGGTCTACAAAACAC |
| AT1G53310 (*AtPPC1*) qRT-PCR RP | AGCATGTGTTCAATGATCTCGA |
| AT2G42600 (*AtPPC2*) qRT-PCR LP | ACGTTCTTGTACCATTCCCTAA |
| AT2G42600 (*AtPPC2*) qRT-PCR RP | AACAACAATAGAATAACACAAGAA |
| At3G14940 (*AtPPC3*) qRT-PCR LP | ATCAGCACAAGAGCTCGTCA |
| At3G14940 (*AtPPC3*) qRT-PCR RP | ACCGGTGTTTTGCAATCCTG |
| AT1G65930 (cytosolic ICDH) qRT-PCR LP | TTGTGGAGAGGAGTGTTGAGA |
| AT1G65930 (cytosolic ICDH) qRT-PCR RP | ACCTAAAAGACCCTAATACCAA |
| AT1G54340 (peroxisomal ICDH) qRT-PCR LP | CAGCAGCGTGATGTTTGATTTG |
| AT1G54340 (peroxisomal ICDH) qRT-PCR RP | ACGTAGCCATTTCTGTTGATTG |
| AT5G14590 (mitochondrial ICDH) qRT-PCR LP | CCTGGGAATTGGGAACAATACA |
| AT5G14590 (mitochondrial ICDH) qRT-PCR RP | GTGTTGGATACGAAACTGAAAAC |
| *UBQ5* qRT-PCR LP | AGAAGATCAAGCACAAGCAT |
| *UBQ5* qRT-PCR RP | AGAAGATCAAGCACAAGCAT |
| *NLP1* qRT-PCR LP | TCAAGGAGCCTCTTGTTCTTG |
| *NLP1* qRT-PCR RP | ATGTCGCTTTCACTTTGATGG |
| *NLP2 qRT-PCR LP* | | TGGTACTGGCACTTCCTTCA | | --- | |
| *NLP2 qRT-PCR RP* | | TGATGGTGGTGACTTTGGAG | | --- | |
| *NLP3 qRT-PCR LP* | | TGTTTGGAGATTCGATGCTG | | --- | |
| *NLP3 qRT-PCR RP* | | CGCTTTGCGATCTCTCTTCT | | --- | |
| *NLP4 qRT-PCR LP* | | TCACAACAAGCCAAGACCAC | | --- | |
| *NLP4 qRT-PCR RP* | | GGGTTTCACTGGAGCAACAT | | --- | |
| *NLP5 qRT-PCR LP* | | ATTCACCACCTGCAAAGTCC | | --- | |
| *NLP5 qRT-PCR RP* | | TATCCGTGGGATCTTCCTTG | | --- | |
| *NLP6 qRT-PCR LP* | | CTCACCAGAGTCGGTAAGCA | | --- | |
| *NLP6 qRT-PCR RP* | | CGGAGACCCATTCTCTATCG | | --- | |
| *NLP8 qRT-PCR LP* | | CAGCGAGAGTGGATCAACAA | | --- | |
| *NLP8 qRT-PCR RP* | | CAACTGATGGCTCGAACTTG | | --- | |
| *NLP9 qRT-PCR LP* | | AATCGAGCGAGGTAATGGAA | | --- | |
| *NLP9* qRT-PCR RP | GAACTTCCCAGCAAAACTGC |
| *NtNRT2.1* qRT-PCR LP | CGGAGGATTTTCTTCAGATTAC |
| *NtNRT2.1* qRT-PCR RP | TGGGCAGAGGATTTGAACGG |
| *NtGS2* qRT-PCR LP | TTCCAGCATAGCATTACCAAGT |
| *NtGS2* qRT-PCR RP | GCATCTGTTGAGCTCTTTGTC |
| *NtGln1-5* qRT-PCR LP | ATTCTGCTGTTTTCCTGGTTTG |
| *NtGln1-5* qRT-PCR RP | ACACCATTGCCAAATCACAGG |
| *NtNii1* qRT-PCR LP | AGTTGGTGATTTTGCTGTGTTCA |
| *NtNii1* qRT-PCR RP | CTCACGTAGGTAGAAATGTTTGC |
| *NtNii2* qRT-PCR LP | GGACTGCTAAGCTTCATGCAACA |
| *NtNii2* qRT-PCR RP | TTATCCCTTGTCTGAATTTTTCC |
| *NtNii4* qRT-PCR LP | CCAAATCATAAAAGATTTTCAAAG |
| *NtNii4* qRT-PCR RP | CTCTAACCTCTCCGATGCTA |
| *NtUbc2* qRT-PCR LP | CTGGACAGCAGACTGACATC |
| *NtUbc2* qRT-PCR RP | CAGGATAATTTGCTGTAACAGATTA |
| *NLP7* RT-PCR LP | GTTTTTCTTTAGACCGCCACC |
| *NLP7* RT-PCR RP | AAGAATCAACCGAACAACACG |
| *Ntβ-actin* RT-PCR LP | GATCTTGCTGGTCGTGATCT |
| *Ntβ-actin* RT-PCR RP | GCGCCACCACCTTGATCTT |
| *NLP7-*attb*-*LP | GGGGACAAGTTTGTACAAAAAAGCAGGCTATGTGCGAGCCCGATGATAATTCC |
| *NLP7-*attb*-*RP | GGGGACCACTTTGTACAAGAAAGCTGGGTTCACAATTCTCCAGTGCTCTCGC |
| *NLP7-*attb*-*RP2 | GGGGACCACTTTGTACAAGAAAGCTGGGTCGCAATTCTCCAGTGCTCTCGCA |
